# Forefoot plantar multilobular noninfiltrating angiolipoma: a case report and review of the literature

**DOI:** 10.1186/1477-7819-6-11

**Published:** 2008-01-30

**Authors:** Theodoros B Grivas, Olga D Savvidou, Spyridon A Psarakis, Georgia Liapi, George Triantafyllopoulos, Ioannis Kovanis, Panagiotis Alexandropoulos, Vasiliki Katsiva

**Affiliations:** 1 Orthopaedic and Pathology department, "Thriasio" General Hospital, G. Gennimata Avenue, Magula, 19600 Greece; 2 Department of Radiology, General Hospital of Nikea-Pireus, Greece

## Abstract

**Background:**

Soft tissue tumors of the feet are uncommon and there have been very few reports of large series in the literature. These tumors continue to present the clinician with one of the most difficult problems in medicine.

**Case presentation:**

We present a case of a large multilobular noninfiltrating angiolipoma at the plantar surface of the forefoot. Only three cases occurring at the foot have been previously described. We report this new case due to unusual location of the tumor, the long duration (25 years) of its existence and the unique surgical approach for the tumor excision.

**Conclusion:**

Surgical excision is the treatment of choice and adjuvant radiotherapy is indicated in select cases.

## Background

Benign lipomatous lesions involving soft tissue are common musculoskeletal masses (almost 50% of all soft-tissue tumors) though they are rare in the foot. They are classified into nine distinct diagnoses: lipoma, lipomatosis, lipomatosis of nerve, lipoblastoma or lipoblastomatosis, angiolipoma, myolipoma of soft tissue, chondroid lipoma, spindle cell lipoma and pleomorphic lipoma, and hibernoma [1].

Angiolipomas are benign neoplasms and have been first described by Bowen in 1912 [2], but were first established as a distinct entity in 1960 by Howard and Helwig [3]. The presence of fibrinous microthrombi is a distinctive feature that differentiates angiolipomas from other lipomas. Sometimes the tumor may be more aggressive and invade the contiguous bone and adjacent soft tissues [4]. We report here a case of angiolipoma of the foot.

## Case presentation

**Figure 1:**
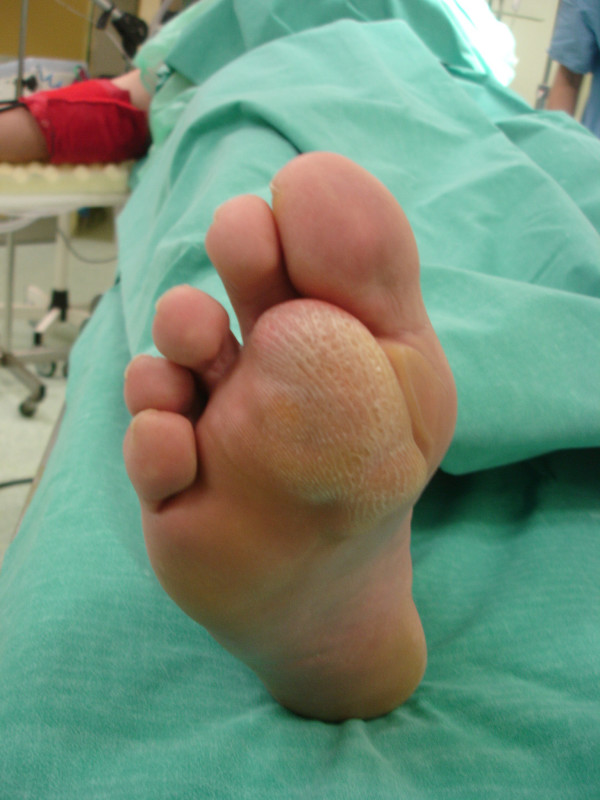
The soft nodular mass at the plantar surface of the forefoot.

A 47-year-old man was admitted to our department with a soft nodular mass at the plantar surface of the forefoot (figure 1). He complained of disabling and painful gait until he was unable to walk and had difficulty putting his shoes on. The patient noticed for the first time the nodule 25 years ago but during the preceding 12 months the size of the nodule had increased markedly.

Physical examination revealed a tender soft-solid nodule. A corn was developed at the overlying skin. No tingling or numbness was present. Neurological consultation was negative. Past medical and familiar history, as well as general examination was negative.

**Figure 2:**
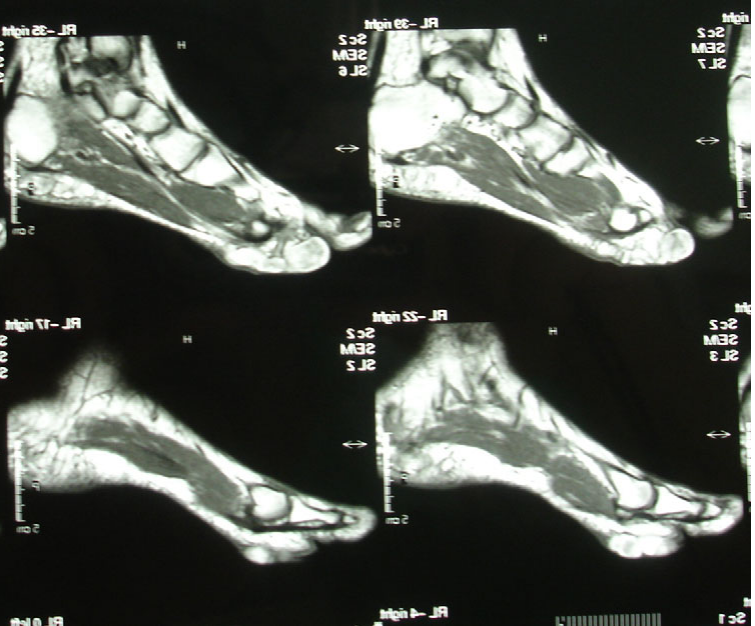
Magnetic resonance imaging (MRI) revealed a well-defined mass located at the plantar forefoot with no apparent bone infiltration.

**Figure 3:**
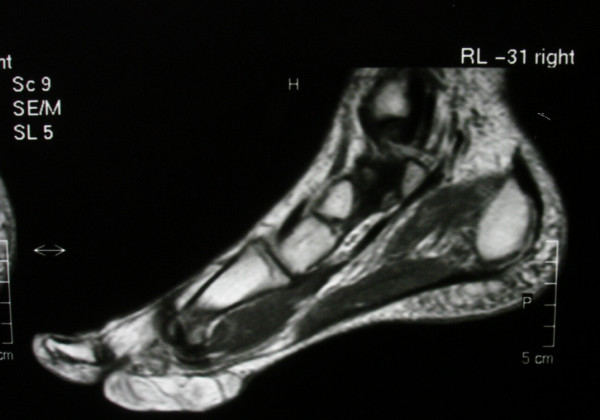
The sagittal T1-weighted image. A lobulated, encapsulated, fatty mass with multiple hypointense nodules and septa in the subcutaneous layer of the forefoot, underneath the plantar aponeurosis.

**Figure 4:**
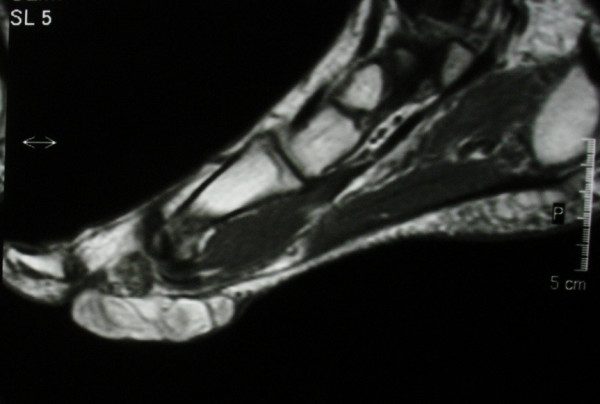
The corresponding sagittal T1-weighted contrast enhanced image. The non-fatty component does not show any apparent enhancement.

**Figure 5:**
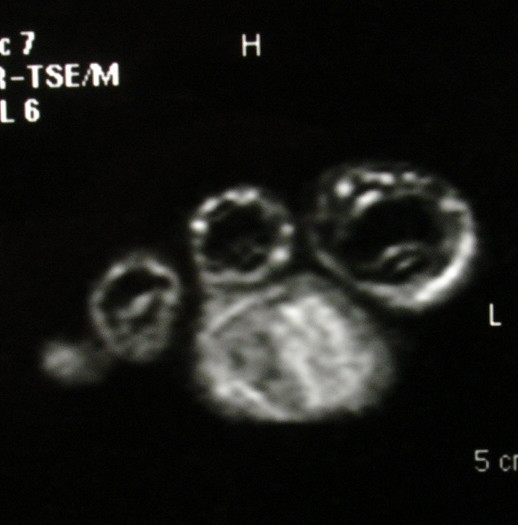
The coronal STIR image through the phalanges. It showed signal suppression of the fatty component and high intensity of the non-fatty component.

Radiographs of the foot and computer tomography (CT) demonstrated a soft-tissue lesion with no osseous involvement. Magnetic resonance imaging (MRI) revealed a well-defined mass located at the plantar forefoot with no apparent bone infiltration, (figure 2). The sagittal T1-weighted image revealed a lobulated, encapsulated, fatty mass (signal intensity identical to subcutaneous fat) with multiple hypointense nodules and septa in the subcutaneous layer of the forefoot, underneath the plantar aponeurosis, (figure 3). The corresponding sagittal T1-weighted contrast enhanced image, revealed that the non-fatty component does not show any apparent enhancement, (figure 4). Finally the coronal STIR image through the phalanges showed signal suppression of the fatty component and high intensity of the non-fatty component, (figure 5). The above assessment was not diagnostic for the pathology, although the duration and the rough imaging of the nodule were not implicating a malignancy.

**Figure 6:**
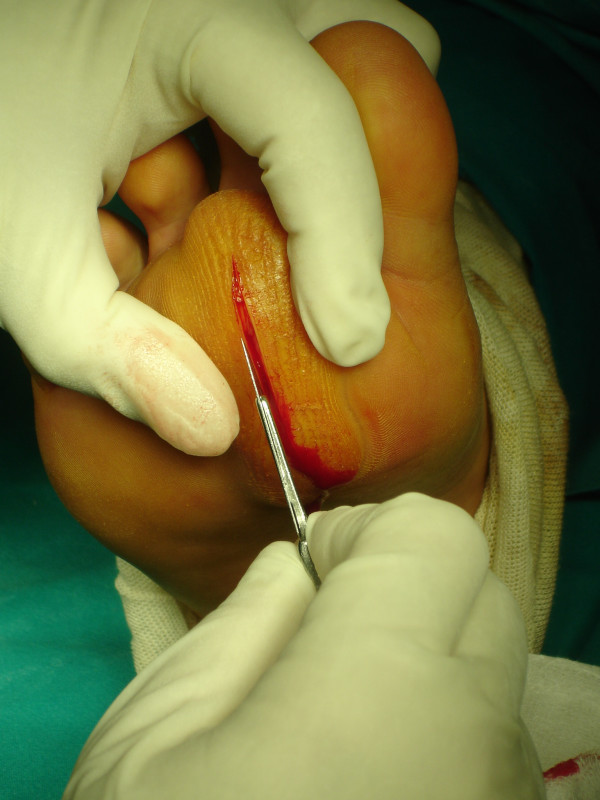
The nodule was excised via a plantar approach using a longitudinal incision dictated by the morphology of the corn.

**Figure 7:**
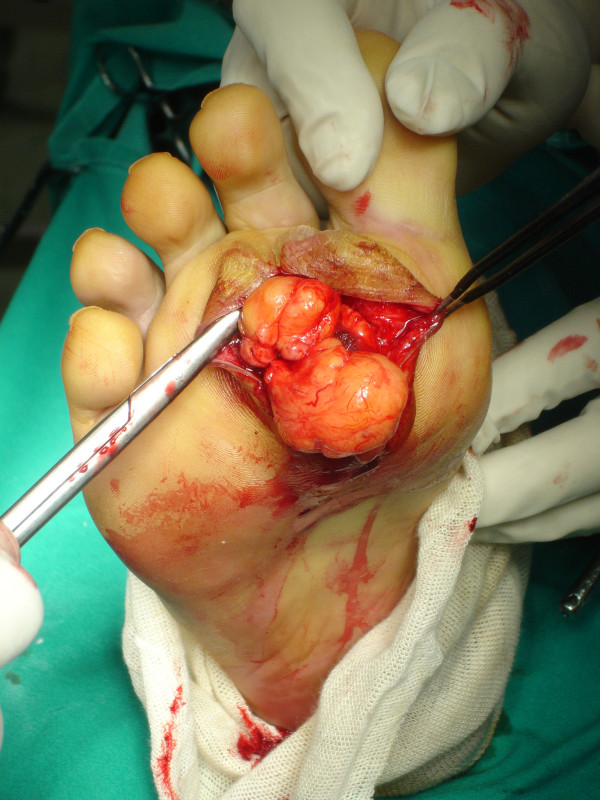
The mass was multilobular having a vascular pedicle which was cauterized.

**Figure 8:**
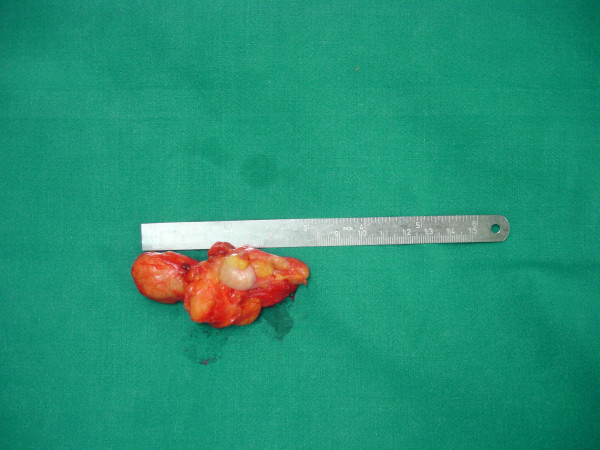
Macroscopically the nodule measuring 7 × 4 × 4 cm and it was encapsulated.

**Figure 9:**
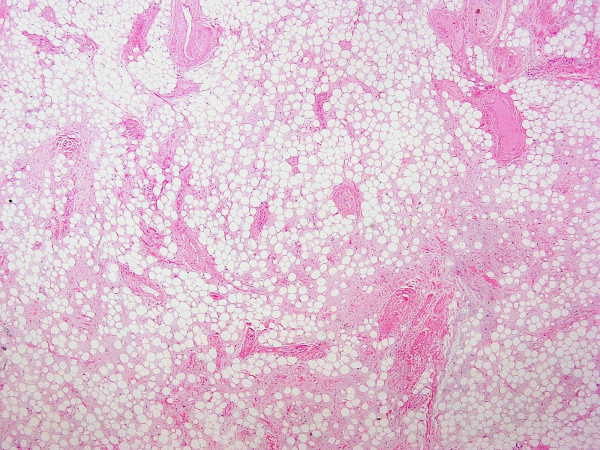
Panoramic view (×4) depicting mature adipose and proliferated vascular tissue.

**Figure 10:**
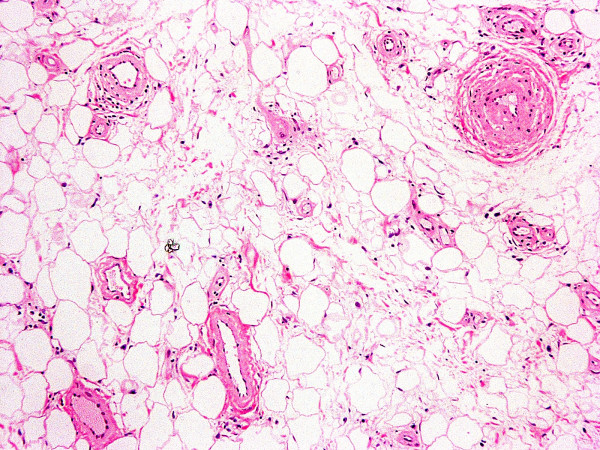
(×10) The mass was comprised of mature adipose and proliferated vascular tissue.

**Figure 11:**
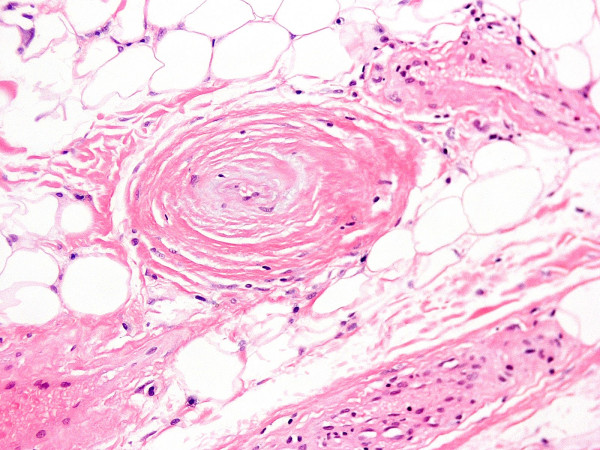
(×20) Thick-walled vessel with collagen deposition and obstruction of the lumen.

**Figure 12:**
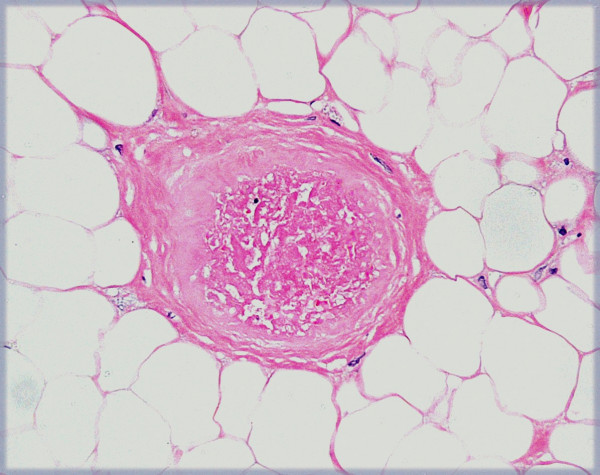
(×20) Very few capillaries demonstrated fibrin thrombi.

**Figure 13:**
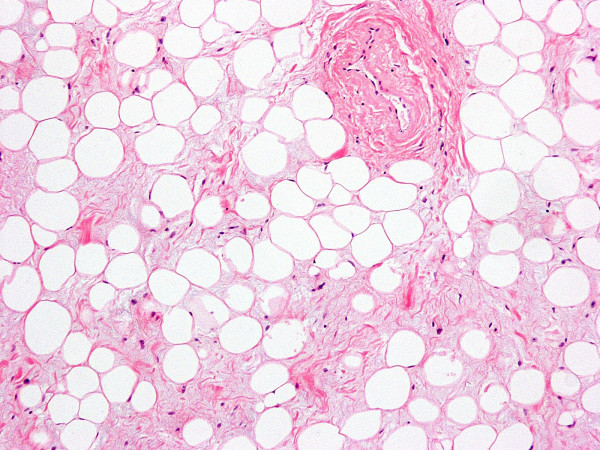
(×10) Degenerative lesions of the adipose tissue.

Marginal surgical excision was performed. The nodule was excised via a plantar approach using a longitudinal incision dictated by the morphology of the corn (figure 6). The location of the presented lesion warranted the use of a plantar approach. Macroscopically the nodule measuring 7 × 4 × 4 cm was encapsulated and multilobular having a vascular pedicle which was cauterized, (figure 7, 8). The mass was totally resected without the need to sacrifice the surrounding structures. The cut surface was solid and yellow with a reddish tinge. In the report describing the pathological examination, it was written the following: "*Gross pathology*: The specimen 7 × 5 × 2 cm. with ill defined margins was yellowish and elastic in consistency. *Histologically*: the mass was comprised of mature adipose and proliferated vascular tissue in various proportion from field to field with no signs of atypia in either of the two components, (Figure 9, 10). Many vessels were thick-walled with collagen deposition which caused obstruction of their lumens (figure 11), while very few capillaries demonstrated fibrin thrombi (figure 12). Adipose tissue showed degenerative lesions with focal deposition of acidic mucopolysaccharides (figure 13). Focal fibrosis and plenty of mast cells were also detected in the interstitial stroma.

The final histologic diagnosis was benign noninfiltrating angiolipoma. The patient's postoperative course was uncomplicated. At the 12-month follow-up no evidence of local recurrence was noticeable.

## Discussion

The pathogenesis of angiolipomas is unknown. They may result from abnormal development of the primitive, pluripotential mesenchymal cells from which adipose tissue and vascular endothelium arise or may be hamartomatous in nature [5]. Other proposed etiologic possibilities include fatty degeneration of a central hemangioma or vascular proliferation of a congenital lipoma [6,7].

On physical examination, angiolipomas usually present as tender, subcutaneous nodules of white adipose tissue. They are rarely associated with overlying skin discoloration. Angiolipoma is a rare variant of lipoma and they occur in the extremities in the spinal axis and in the neck and head [4-6,8,9]. Only three cases occurring at the foot have been previously described [4,8,9]. The most common symptom is a constant, dull pain with associated neuropathies secondary to vascular engorgement and edema, which can lead to compression of the adjacent neural tissue [10,11]. Our patient had a tender, semi-mobile nodule at the plantar surface of the forefoot.

The diagnosis of angiolipoma can be aided by computed tomography (CT) or magnetic resonance imaging (MRI). On contrast-enhanced studies, angiolipomas demonstrate a marked enhancement as a result of their intense vascularity. Noncontrast studies demonstrate the homogenous low attenuation of a typical lipoma [11]. In our patient, MRI detected a well-defined lesion with no infiltration into adjacent tissues. In our case, also, the presence of many thick-walled vessels and the degenerative lesions of the adipose tissue to our opinion can be explained on the bases of the "age", (long duration), of the neoplasm and its location, which caused mechanical pressure. Beside this estimation the mast cells, which observed in high numbers, play a role to the consistency of the intermediate stroma.

The main challenge of these otherwise benign tumors is first to establish a correct diagnosis. They belong to a wider spectrum ranging from benign pure lipomas, composed of adipose tissue, to benign pure angiomas, composed of vascular tissue. They probably lie in the middle of this spectrum and according to the relative percentages of adipose and vascular tissues, can be divided as lipomatous or angiomatous types [8,9].

Although a presumptive diagnosis is typically made clinically, these tumors with atypical clinical features may require radiological consultation. Difficulty arises when radiographic features are not typical of lipoma. Radiological evaluation is diagnostic in up to 71% of cases. These lesions are identical to subcutaneous fat on computed CT and MRI images [1]. MRI could be a useful tool to diagnose local areas of infiltration [4].

Histopathologically angiolipomas are characterized by mature adipose tissue containing copious vascular elements that vary from sinusoids, thin-walled vessels or thick-walled vessels with proliferation of the smooth muscle layer [12]. Mitotic figures are infrequent and malignant changes have not been identified [13]. They vary in color from whitish-yellow to a grayish-purple. Immunohistochemistry, if histology is not helpful, can be of some help in the final diagnosis.

Based on studies by Dionne [14] and Lin [13], angiolipomas are subdivided into two histological types: infiltrating and noninfiltrating. Infiltrating angiolipomas are characteristically not encapsulated, and they infiltrate into surrounding tissue. Their clinical behavior is similar to that of hemangiomas. Infiltrating angiolipomas are usually diagnosed in older patients. The vast majority occur in the lower extremities or in the paraspinal region, which can lead to muscular pain and neural deficits [6,11,15]. In their study of 459 lipomas, Lin and Lin [13] found that 25 (5.4%) met the criteria for angiolipoma. Two of the 25 angiolipomas were microscopically unencapsulated and showed some degree of infiltration into adjacent tissues. Noninfiltrating, or circumscribed, angiolipomas are encapsulated lesions limited to the subcutaneous compartment. Their size almost never exceeds 4 cm. These lesions are more common in young people, and they are equally distributed between the sexes.

Although angiolipomas are benign lesions sometimes they can be more aggressive and invade the contiguous bone and adjacent soft tissues [16,17]. Contrary to lipomas and angiomas, the possibility to infiltrate bone and bone marrow renders them more susceptible to local recurrence [4]. In these cases, only bone amputation or postoperative radiotherapy can provide a definitive cure [8,9].

Differentiation of angiolipomas from liposarcomas based on imaging features is not possible some times necessitating surgical resection for definitive histological diagnosis [18]. The differentiation is based on cellular atypia, mitotic figures, and cellular pleomorphism, which is seen with malignant lesions. In addition, the lipocytes of liposarcoma resemble embryonic adipose tissue and the vasculature of liposarcoma contains only capillaries, and the veins are seen within the angiolipoma. Differentiation of angiolipomas from others lipoma variants (lipomatosis, myolipoma, chondroid lipoma, hibernoma, spindle cell lipoma, atypical lipoma, pleomorphic lipoma, lipoblastoma) and understanding the spectrum of appearances of the various benign musculoskeletal lipomatous lesions improves radiological assessment and is vital for optimal patient management. Lipomatosis represents a diffuse overgrowth of mature fat affecting subcutaneous tissue, muscle or nerve, and imaging is needed to evaluate lesion extent. Lipoblastoma is a tumor of immature fat occurring in young children, and imaging features may reveal a mixture of fat and nonadipose tissue. Angiolipoma, myolipoma, and chondroid lipoma are rare lipomatous lesions that are infrequently imaged. Spindle cell and pleomorphic lipoma appear as a subcutaneous lipomatous mass in the posterior neck or shoulder, with frequent nonadipose components. Hibernoma appears as a lipomatous mass with serpentine vascular elements.

Benign lipomatous lesions affecting bone, joint, or tendon sheath include intraosseous lipoma, parosteal lipoma, liposclerosing myxofibrous tumor, discrete lipoma of joint or tendon sheath, and lipoma arborescens. Intraosseous and parosteal lipoma have a pathognomonic CT or MRI appearance, with fat in the marrow space or on the bone surface, respectively. Liposclerosing myxofibrous tumor is a rare intermixed histological lesion commonly located in the medullary canal of the intertrochanteric femur. Benign lipomatous lesions may occur focally in a joint or tendon sheath or with diffuse villonodular proliferation in the synovium (lipoma arborescens) and are diagnosed based on location and identification of fat.

The treatment of both infiltrating and noninfiltrating angiolipomas is total surgical excision. The infiltrating type of lesion is associated with more treatment difficulties. These lesions have been reported to recur after surgical excision in 35 to 50% of cases [14]. Wide local excision with free margins is the preferred surgical procedure; in cases of inadequate excision, radiation therapy is necessary [6,11]. For noninfiltrating angiolipomas, simple excision is curative because these lesions have no tendency to recur following surgical removal. In our patient marginal surgical excision using a longitudinal incision was performed and after one-year of follow-up the patient showed no signs of recurrence.

## Competing interests

The author(s) declare that they have no competing interests.

## Authors' contributions

**TBG **was the principal investigator of the study, operated upon the patient, conducted the collection of data and involved in drafting the article. **ODS **involved in drafting the article and involved in collection of the literature, **SAP **helped in manuscript drafting and in the collection of the literature, the **GL **performed the pathological examination, wrote the report and involved in drafting the article and **GT, IK, PA **were involved in collection of the literature and drafting of manuscript. **VK **made the radiological diagnosis and report. All the authors read and approved the final manuscript.
